# Mitral Transcatheter Edge‐to‐Edge Repair in Patients With Advanced Heart Failure: A Single‐Center Experience and Insights Into Anatomical and Clinical Determinants of Procedural Complexity

**DOI:** 10.1002/ccd.70574

**Published:** 2026-03-23

**Authors:** Pedro Castilhos de Freitas Crivelaro, Laura Hastenteufel, Guilherme Pinheiro Machado, Felipe Fuchs, Antonio Pinotti, Marco Vugman Wainstein, Nadine Clausell, Lívia Adams Goldraich, Felipe Homem Valle

**Affiliations:** ^1^ Interventional Cardiology Hospital de Clínicas de Porto Alegre Porto Alegre Rio Grande do Sul Brazil; ^2^ Advanced Heart Failure and Heart Transplant Program Hospital de Clínicas de Porto Alegre Porto Alegre Rio Grande do Sul Brazil; ^3^ Division of Cardiology Hospital de Clínicas de Porto Alegre Porto Alegre Rio Grande do Sul Brazil

**Keywords:** advanced heart failure, MitraClip, mitral regurgitation

## Abstract

**Background:**

Advanced heart failure (HF) remains a clinical challenge, and mitral transcatheter edge‐to‐edge repair (M‐TEER) has emerged as a potential bridging strategy.

**Aims:**

To describe the clinical outcomes of M‐TEER in a single‐center cohort of patients with advanced HF and to identify anatomical and clinical features potentially associated with increased procedural complexity.

**Methods:**

This retrospective case series included 13 consecutive patients with advanced HF and moderate‐to‐severe or severe mitral regurgitation (MR) who underwent M‐TEER using the MitraClip system. Clinical, echocardiographic, and hemodynamic data were analyzed.

**Results:**

The median age was 58 years (range 39–64), and the median EuroSCORE II was 10.4% (3.86–15.07). All patients had reduced left ventricular (LV) ejection fraction (median 22%) and enlarged LV dimensions (end‐systolic diameter > 70 mm in 38%). The mean tenting height was 15.4 ± 2.5 mm. Five patients (38.4%) required continuous inotropic support. Procedural success was achieved in all cases, with no periprocedural or 30‐day mortality. Over a median follow‐up of 287 days (171–483), four patients (30.7%) died, one (7.6%) underwent urgent heart transplantation, and two (15.3%) had elective transplantation. Four of five patients on inotropes (80%) were weaned off support. At 1 year, 61.5% of patients remained free from the composite endpoint of death, HF hospitalization, urgent transplant, or LV assist device implantation.

**Conclusions:**

M‐TEER was feasible and safe in selected patients with advanced HF. This cohort presented anatomical and clinical features that may have contributed to greater procedural complexity.

## 1 Introduction

Advanced heart failure (HF) is characterized by refractoriness to pharmacological and non‐pharmacological treatments, and, at this stage of the disease, heart transplant (HTx) and left ventricular assist devices (LVAD) are the main therapies available with a focus on prolonging life expectancy and improving quality of life [1]. However, the increasing number of patients with advanced HF and a fairly stable and limited availability of donor organs create a widening imbalance, resulting in prolonged waiting time to transplant and elevated mortality among wait‐listed patients [2]. In this scenario, bridging strategies have been employed to improve clinical status, refrain, and/or prevent end‐organ compromise.

Functional mitral regurgitation (FMR) is often present in advanced HF, and it is associated with worse prognosis and increased mortality [3]. In this scenario, mitral transcatheter edge‐to‐edge repair (M‐TEER) was evaluated as a therapeutic strategy in an international registry showing that it is a safe and effective bridging strategy in selected patients with advanced HF and severe FMR [4].

Anatomical criteria considered ideal for M‐TEER have been mainly derived from the COAPT trial, which selected patients with secondary mitral regurgitation (MR) and optimized guideline‐directed medical therapy (GDMT). These criteria include a posterior leaflet length > 10 mm, coaptation depth (tenting height) < 10 mm, mitral valve area > 4.0 cm², and central regurgitant jet (A2/P2). Additional favorable features include an end‐systolic LV diameter ≤ 70 mm and systolic pulmonary artery pressure < 70 mmHg. Patients with extensive annular or leaflet calcification, complex jet morphology, multiple jets, or short/tethered posterior leaflets were generally excluded, given the higher likelihood of suboptimal results or residual mitral stenosis after clip implantation [5]. However, such ideal anatomical conditions are frequently absent in patients with advanced HF. In this population, FMR often coexists with significant ventricular dilatation and dysfunction, resulting in anatomical distortions such as increased tenting height, reduced leaflet mobility, small mitral valve areas, and asymmetric leaflet tethering [6]. These patients may also present with elevated pulmonary pressures and advanced remodeling, complicating M‐TEER planning and execution [7].

In this article, we report a cohort of patients with advanced HF treated with a MitraBridge strategy, highlighting anatomical challenges associated with M‐TEER in this high‐risk population with a detailed assessment of leaflet characteristics, ventricular geometry, and mitral valve morphology. In addition, we report clinical outcomes at 30 days and 1 year, providing insights into real‐world decision‐making and procedural feasibility beyond traditional COAPT‐like anatomy.

## 2 Methods

### 2.1 Study Design

This retrospective case series describes the experience of M‐TEER in selected patients with advanced HF who were being considered for advanced therapies and/or were listed for HTx and presented with moderate to severe or severe FMR in a regional reference center.

### 2.2 Population

All consecutive patients with advanced HF who underwent M‐TEER were included and classified according to a pre‐specified therapeutic strategy: bridge‐to‐decision (BTD), for potential candidates for heart transplantation with pending final decision regarding the timing to be listed; bridge‐to‐transplantation (BTT), for patients already on the waiting list, in whom timely heart transplantation was unlikely and with ongoing deteriorating clinical status; bridge‐to‐candidacy (BTC), for patients with potentially reversible contraindications for transplantation, such as severe pre‐capillary pulmonary hypertension; destination therapy (DT), for those who were not candidates for heart transplantation, because of non‐reversible contraindications. Since advanced HF substantially increases surgical risk, all patients were deemed not to be candidates for mitral valve surgery. All patients underwent M‐TEER using the MitraClip system (Abbott Laboratories, Chicago, U.S.). MR, as well as other echocardiographic parameters, were assessed as per the American Society of Echocardiography and the European Association of Echocardiography guidelines' recommendations [8, 9]. Perioperative aspects were planned for each individual patient during case discussions, including an anesthetist, an interventionist, and an HF specialist. Oral vasodilators were held to avoid vasoplegia. A more liberal use of inotropes with slow postprocedural weaning was allowed to prevent from afterload mismatch (AM). A conservative approach aiming at MR improvement in two grades was planned to avoid extensively prolonged procedures for selected patients.

### 2.3 Study Endpoints

Patients were followed during their index hospitalizations in which M‐TEER was performed and then throughout periodic visits to the HF Clinic following discharge. We reported echocardiographic, hemodynamic, and procedural characteristics. The clinical outcomes reported were: 30‐day survival, 1‐year all‐cause mortality, incidence of HTx, rates of rehospitalization for HF, and weaning of inotropes. We also reported the clinical status of patients at the time of the last available follow‐up. A transthoracic echocardiogram (TTE) was performed prior to hospital discharge and thereafter according to clinical indication in the outpatient setting. Mitral Valve Academic Research Consortium criteria were used to define procedural success [10].

### 2.4 Statistical Analysis

In this descriptive report, categorical variables are presented as absolute numbers and percentages, and quantitative variables, as mean ± standard deviation or median and interquartile range, as appropriate. Given the small sample size (*n* = 13), most variables were reported as medians and interquartile ranges, while means and standard deviations were used for normally distributed parameters. Survival free from the composite endpoint (all‐cause death, HF hospitalization, urgent HTx, or LVAD implantation) was calculated as the proportion of patients without any of these events during the first 12 months of follow‐up. Pre and postprocedural V wave measurements were compared using a paired *t*‐test. All hypothesis tests were two‐sided, with a significance level set at 0.05. Statistical analyses were performed using SPSS Statistics for Windows, Version 29.0 (IBM Corp., Armonk, NY, USA).

## 3 Results

### 3.1 Clinical Characteristics

Thirteen patients with advanced HF who underwent M‐TEER at our center between February 2021 and 2025 were included. Baseline clinical characteristics are summarized in Table 1. The median EuroSCORE II was 10.4% (25th–75th percentile: 3.86–15.07). Most patients were male (*n* = 8, 61.5%) and had non‐ischemic cardiomyopathy (*n* = 7, 53.8%), with a median age of 58 years (25th–75th percentile: 39–64). All patients were receiving maximally tolerated GDMT optimized by a heart‐failure specialist; however, target doses could not be achieved in most cases because of advanced disease and limited drug tolerance. At the time of the procedure, five patients (38.4%) were receiving long‐term continuous intravenous inotropic support for clinical indications (INTERMACS profile ≤ 3). In addition, one patient (7.7%) required short‐term intravenous inotropic support limited to the periprocedural period. MR was severe in eight patients (61.5%) and moderate‐to‐severe in five patients (38.4%). At the time of the M‐TEER procedure, patients were stratified into four predefined therapeutic strategies based on their transplant candidacy and clinical status. The BTD group was the most represented, encompassing six patients (46.1%), four patients (30.7%) were categorized as DT, two patients (15.3%) were in the BTT group, and one patient (7.6%) was assigned to the BTC group.

**Table 1:** Baseline clinical characteristics.

Clinical characteristics	Overall (*n* = 13)
Age, years	58 (39−64)
Male sex	8 (61.5)
Diabetes	3 (23)
CKD	7 (53.8)
Atrial fibrillation	5 (38.4)
COPD	0
NYHA III/IV	13 (100)
Ischemic MR	6 (46.1)
Non‐ischemic MR	7 (53.8)
INTERMACS	
1–2	1 (7.6)
3–4	7 (53.8)
5–6	5 (38.4)
7	0
Inotropes	5 (38.5)
Euroscore II	10.4 (3.86–15.07)
Past medical history	
Previous AMI	6 (46.1)
Previous PCI	5 (38.4)
Previous CABG	1 (7.6)
Previous stroke	2 (15.3)
HF hospitalization within 12 months	11 (84.6)
GDMT at baseline	
ACE‐I or ARB	1 (7.6)
ARNI	5 (38.4)
Beta‐blocker	8 (61.5)
MRA	10 (76.9)
SGLT2 inhibitors	7 (53.8)
Loop diuretics	13 (100)
Digoxin	11 (84.6)
ICD	10 (76.9)
CRT	2 (15.3)

*Note:* Data are presented as absolute numbers and percentages (for categorical variables) and median value and 25th and 75th percentiles (for continuous variables).

Abbreviations: ACE‐I, angiotensin‐converting enzyme inhibitors; AMI, acute myocardial infarction; ARB, angiotensin receptor blocker; ARNI, angioten‐sin receptor neprilysin inhibitor; BTC, bridge to candidacy; BTD, bridge to decision; BTT, bridge to transplant; CABG, coronary artery bypass grafting; CKD, chronic kidney disease; COPD, chronic obstructive pulmonary disease; CRT, cardiac resynchronization therapy; DT, destination therapy; GDMT, guideline‐directed medical therapy; HF, heart failure; ICD, implantable cardioverter defibrillator; INTERMACS, Interagency Registry for Mechanically Assisted Circulatory Support; MR, mitral regurgitation; MRA, mineralocorticoid receptor antagonist; NYHA, New York Heart Association; PCI, percutaneous coronary intervention.

### 3.2 Echocardiographic Characteristics and Hemodynamic Variables

Baseline transthoracic echocardiography revealed that the median effective regurgitant orifice area (EROA) was 0.40 cm² (25th–75th percentile: 0.26–0.46 cm²), and the median regurgitant volume was 33 mL/beat (25th–75th percentile: 29–42 mL/beat). Left ventricular ejection fraction (LVEF) was markedly reduced, with a median of 22% (25th–75th percentile: 20%–30%), and all patients exhibited substantial LV dilation (mean LV end‐diastolic diameter: 70.3 ± 8.6 mm). Left ventricular end‐systolic diameter (LVESD) higher than 70 mm was present in 38% of cases. In addition, right ventricular involvement was frequently observed: the mean basal right ventricular diameter was 45 ± 9 mm, the mean tricuspid annular plane systolic excursion (TAPSE) was 14 ± 3 mm, and the median fractional area change (FAC) was 24% (25th−75th percentile: 20.5%−31.5%). Detailed echocardiographic and hemodynamic data are presented in Table 2. Regarding features of procedural complexity, the average tenting height was 15.4 ± 2.5 mm. Additionally, 23% of the patients exhibited eccentric MR jets.

**Table 2:** Baseline echocardiographic and right heart catheterization characteristics.

Echocardiographic	Overall (*n* = 13)
LVEF (%)	22 (20–30)
LVEDV (mL)	247 (194–307)
LVESV (mL)	201 (130–264)
LVEDD, mm	70.3 ± 8.6
LVESD, mm	63 ± 10.1
LVESD > 70 mm	5 (38)
LAVi, mL/m^2^	69 (60–75)
sPAP, mm Hg^a^	56 (44–59.5)
TAPSE (mm)^a^	14 ± 3
FAC (%)	24 (20.5–31.5)
Tricuspid regurgitation > 2	6 (46.1)
Basal RVD (mm)	45 ± 9
MR characteristics	
Moderate to severe	5 (38.4)
Severe	8 (61.5)
EROA (mm^2^)	0.4 (0.26–0.47)
RVol (mL/beat)	33 (29–42)
Central regurgitation (A2–P2)	10 (76.9)
Posterior leaflet (mm)	13.2 ± 1.1
Tenting height (mm)	15.4 ± 2.5
Proportionate MR^b^	5 (38.4)
Right heart catheterization	
Cardiac index (L/min/m^2^)	2.3 (2–2.6)
CVP (mm Hg)	6 (5–10)
Systolic PAP (mm Hg)	50 (40–60)
Diastolic PAP (mm Hg)	25 (17–30)
Mean PAP (mm Hg)	34 (26–39)
Wedge pressure (mm Hg)	23 (16–30)
V wave‐wedge (mmHg)	35 (25–40)
PRV (WU)	2.2 (2–2.6)

*Note:* Data are presented as absolute numbers and percentages (for categorical variables) and mean value § SD or median value and 25th and 75th percentiles (for continuous variables).

Abbreviations: CVP, central venous pressure; EROA, effective regurgitant area; FAC; fractional area change; LAVi, left atrial volume index; LVEDD, left ventricular end‐diastolic diameter; LVEDV, left ventricular end‐diastolic volume; LVEF, left ventricular ejection fraction; LVESD, left ventricular end systolic diameter; LVESV, left ventricular end systolic volume; PAP, pulmonary artery pressure; PVR, pulmonary vascular resistance; RVD, right ventricular diameter; Rvol, regurgitant volume; sPAP, systolic pulmonary artery pressure; TAPSE, tricuspid annular plane systolic excursion; WU, Wood unit.

^a^ Data for these parameters were not available for all patients.

^b^ Proportionate MR was defined as the relation EROA/LVEDV < 0.14 [11].

**Figure 1:**
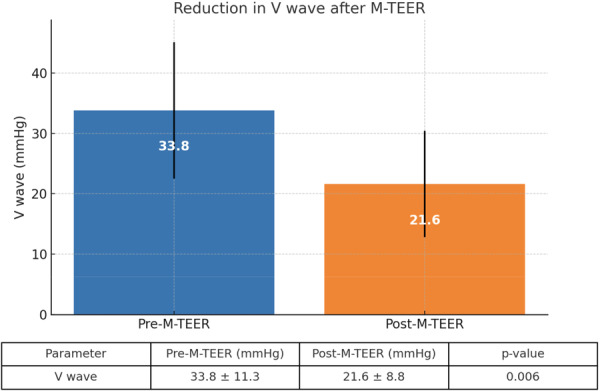
Reduction in V wave post‐M‐TEER. [Color figure can be viewed at wileyonlinelibrary.com]

All patients presented with elevated pulmonary artery wedge pressure, with a mean value of 23 ± 8 mmHg and a mean value of V wave of 33.8 ± 11.3 (Figure 1). Most of them also had reduced cardiac index, with a median value of 2.3 L/min/m² (25th–75th percentile: 2.0–2.6) and elevated mean pulmonary artery pressure, with a median value of 34 mmHg (25th–75th percentile: 26–39).

### 3.3 Procedural Results

Procedural success was achieved in all cases [10]. Most patients required either one (*n* = 7, 53.8%) or two (*n* = 5, 38.4%) clips, and one patient (7.6%) required three clips. Among the 13 patients treated, 11 (84.6%) received a fourth‐generation MitraClip XT Wide device, while two patients (15.4%) were treated with third‐generation devices (NTR or XTR; Abbott Laboratories, Chicago, U.S.) (Table 6), At the end of the procedure, residual MR was ≤ 1+ in eight patients (61.5%) and grade 2+ in five patients (38.5%). No periprocedural or 30‐day deaths were observed. The median procedure time was 188 min (25th–75th percentile: 164.2–203.5). Despite the increased procedural duration, there were no vascular complications or instances of single‐leaflet device attachment (SLDA). There was a pronounced hemodynamic change in the left atrial V wave amplitude, which decreased from a median of 33.8 ± 11.3 to 21.6 ± 8.8 mmHg (*p *= 0.006) (Table 3).

**Table 3:** Procedural outcomes.

Procedural success	13 (100)
Procedural duration (min)	188 (164.2–203.5)
Vascular complication	0
SLDA	0
Number of clips	
0	0
1	7 (53.8)
2	5 (38.4)
> 3	1 (7.6)

*Note:* Data are presented as absolute numbers and percentages (for categorical variables) and median value and 25th and 75th percentiles (for continuous variables).

Abbreviation: SLDA, single‐leaflet device attachment.

### 3.4 Clinical Outcomes

The median follow‐up was 287 days (25th–75th percentile: 171–483). Four patients receiving inotropic support (80%) were successfully weaned off from it. The other patient who remained on inotropic support underwent urgent heart transplantation. Two patients (23%) underwent successful elective heart transplantation at 5 and 19 months after M‐TEER, respectively. At the last follow‐up, the three who underwent heart transplantation remained alive with preserved graft function.

**Figure 2:**
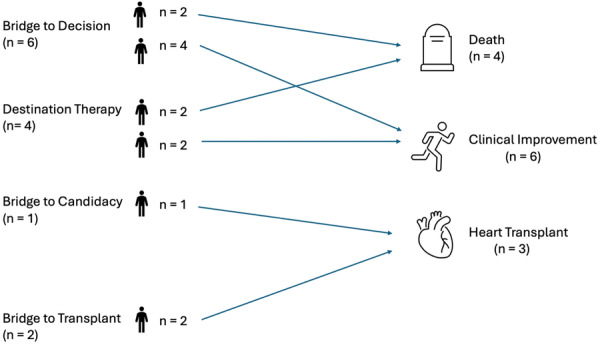
Clinical outcomes according to the therapeutic strategy before M‐TEER. [Color figure can be viewed at wileyonlinelibrary.com]

**Figure 3:**
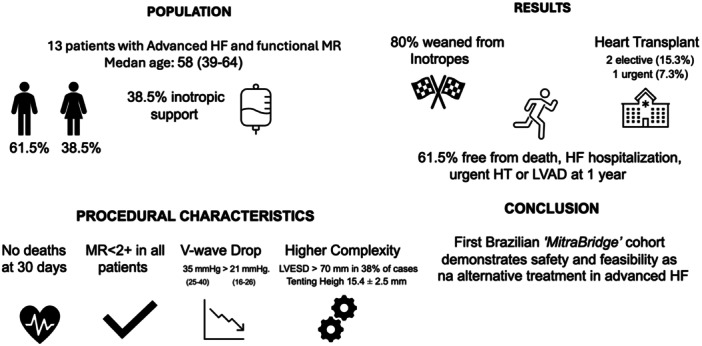
Overview of population, procedural characteristics, and clinical outcomes following M‐TEER in advanced heart failure.

Four deaths (30.7%) were recorded during follow‐up, occurring at a median of 6.5 months (25th–75th percentile: 4.75–9.75) after the procedure: two were attributed to progressive HF and two were classified as sudden cardiac death. There was no LVAD implantation during follow‐up. The median rate of HF hospitalization was 23% with a median of 100 days (25th–75th percentile: 96–128) after the procedure. At 1 year, eight patients (61.5%) were free from the composite endpoint of death, HF hospitalization, urgent HTx, or LVAD implantation (Table 4). Figure 2 illustrates individual postprocedural outcomes stratified by initial therapeutic strategy. Figure 3 summarizes the study population, procedural characteristics, and clinical outcomes.

**Table 4:** Clinical outcomes.

Composite outcome of death, HF hospitalization, urgent transplant, or LVAD implantation	5 (38.4)
Follow‐up (days)	287 (171–483)
30‐day survival	13 (100)
One year of death	3 (23)
Median time to death (days)	214 (160.7–313.5)
Cardiac death	4 (100)
HF death	2 (50)
Sudden death	2 (50)
HF hospitalization	3 (23)
Time from M‐TEER to HF hospitalization	100 (96–128)
Urgent transplant	1 (7.6)
Elective transplant	2 (15.3)
Median time to transplant	177 (130–390)
Weaning from inotropic	4 (80)
Median time to wean off inotropes (days)	9.5 (3.5–21)

*Note:* Data are presented as absolute numbers and percentages (for categorical variables) and median value and 25th and 75th percentiles (for continuous variables).

Abbreviation: HF, heart failure.

## 4 Discussion

This study describes our initial experience with M‐TEER in patients with advanced HF and moderate to severe MR. In this case series, patients were in very limited functional capacity, and in five, under inotropic support. The median EuroSCORE II in our cohort (10.4%) was higher than the median surgical risk reported in the MitraBridge registry, highlighting the increased baseline complexity of our population. Despite that, M‐TEER was safely performed, and procedural success was achieved in all cases. Clinical outcomes of withdrawal of inotropic support, elective rather than urgent transplants, and deferred heart transplantation were observed, suggesting a potential beneficial role for M‐TEER in this setting.

In the largest cohort evaluating M‐TEER in advanced HF, the MITRABRIDGE Registry, 64% of 119 patients remained free from the composite of death, urgent HTx, LVAD implantation, or HF hospitalization at 1 year. Notably, HTx was considered unnecessary (deferred) in 23.5% of patients [2]. In our series, after a median follow‐up of 9 months (25th–75th percentile: 5–15), the 1‐year rate of freedom from death, HF hospitalization, urgent HTx, or LVAD implantation was 61.5%, similar to that reported in the international registry. Additionally, eligibility or safe access to transplant was achieved in three cases (23%), and HTx was ruled out in six patients (46.1%). Two patients (15.3%) subsequently underwent elective HTx, and in both cases, M‐TEER facilitated clinical stabilization and hospital discharge before transplantation. In contrast, 11.9% of patients in MITRABRIDGE ultimately received elective LVAD implantation; the absence of LVAD access in our setting may partly explain the higher proportion of transplants in our cohort.

Historically, skepticism prevailed regarding the benefit of M‐TEER in patients with markedly dilated ventricles, driven by the early concept that proportionate MR signified disease too advanced to derive meaningful reverse remodeling [11]. However, contemporary evidence, mostly provided from international registries, increasingly shows that M‐TEER may confer significant symptomatic relief at short term [12] and may provide sustained clinical benefit in “non‐COAPT” phenotypes [13].

A recent prospective non‐randomized comparison based on anatomical eligibility criteria showed that M‐TEER confers clear clinical superiority over isolated medical therapy in INTERMACS 3–4 patients, enabling successful hospital discharge in most cases, improving tolerance to GDMT, and reducing the incidence of the combined primary endpoint (death, urgent heart transplantation, or LVAD implantation at 12 months) [14]. Consistent with these findings, our cohort demonstrated unequivocal hemodynamic improvement after M‐TEER, supported by two key observations. First, the marked reduction in left atrial V‐wave amplitude reflected immediate atrial unloading and effective mitigation of regurgitant flow. Second, all patients receiving inotropic support were successfully weaned following the procedure, indicating enhanced forward stroke volume and improved circulatory stability. This hemodynamic improvement is particularly valuable in the setting of advanced HF, as these patients often cannot tolerate GDMT due to systemic hypotension and associated renal dysfunction. In this context, M‐TEER may be important in enabling the escalation and optimization of pharmacologic therapy and in facilitating hospital discharge.

In the absence of randomized data and given the heterogeneous inclusion criteria across registries evaluating M‐TEER in advanced HF, a major challenge remains the identification of clinical and anatomical characteristics that reliably predict which patients are most likely to benefit from the procedure. The COAPT Risk Score, a prognostic tool that incorporates clinical, echocardiographic, and treatment‐related variables to estimate the 2‐year risk of death or heart‐failure hospitalization in patients with severe FMR, showed limited performance during external validation, indicating that the idealized COAPT selection criteria may not be applicable to the advanced heart‐failure population [15, 16]. Machine‐learning‐based tools are emerging to phenotype patients with FMR undergoing TEER and to identify high‐risk profiles; for example, the MITRA‐AI study applied unsupervised clustering to delineate distinct clinical phenotypes with significantly different rates of cardiovascular death and heart‐failure hospitalization, findings that were externally validated and may improve patient selection [17].

Right ventricular dysfunction—including RV–pulmonary artery uncoupling—and biventricular impairment have also been shown to predict lower procedural success and poorer clinical outcomes [18, 19]. Our cohort showed a high prevalence of RV dysfunction (median FAC 24%), reflecting the severity of disease in this population. Nutritional status is another clinically important determinant: a large multicenter study reported that approximately 74% of patients undergoing M‐TEER exhibit some degree of malnutrition, and moderate–severe malnutrition independently predicts mortality and HF rehospitalization [20].

Because our cohort was retrospective, no uniform selection criteria were applied prospectively, and all advanced HF cases treated at our center were included. In hindsight, the population represents an exceptionally high‐risk group, characterized by inotrope dependence, right ventricular dysfunction, pulmonary hypertension, markedly dilated ventricles, and anatomically complex mitral valve features. Despite this adverse profile, the outcomes achieved were unexpectedly favorable and comparable to those reported in international registries. These results likely reflect the expertise of a highly experienced multidisciplinary heart team and the benefits of rigorous longitudinal follow‐up and optimized medical therapy.

Despite the higher procedural risk, M‐TEER is also more technically demanding in advanced HF. In our cohort, elevated tenting height (median of 15.4 ± 2.5 mm) and markedly increased LVESD (higher than 70 mm in 38% of cases) were anatomical features observed, and both of which are known to impair leaflet coaptation and increase the complexity of device positioning and effective MR reduction (Table 5). In the context of M‐TEER, a tenting height greater than 10 mm implies apical displacement of leaflet coaptation due to severe subvalvular distortion [5]. This configuration reduces leaflet mobility and limits the ability of the device to achieve adequate leaflet grasping and durable coaptation. These technical limitations increase the likelihood of residual MR—a well‐established predictor of worse clinical outcomes after M‐TEER [21, 22]. This may also help explain why patients with proportionate MR, who typically exhibit more advanced LV disease and more severe geometric distortion, experience poorer results compared with those with disproportionate MR [11].

**Table 5:** Parameters demonstrating higher complexity and risk, if compared with ideal non‐complex M‐TEER criteria.

**Parameter**	**Non‐complex Ideal for M‐TEER (COAPT trial)**	**Advanced HF cohort**
LVESD	≤ 70 mm	> 70 mm in 38% of cases
Tenting height	< 10 mm	15.4 ± 2.5 mm
RV dysfunction	COAPT‐excluded	Median FAC of 24% (20.5%–31.5%)
RV systolic pressure	44.0 ± 13.4	50 (40–60)
EF	31.3 ± 9.1	22 (20–30)
EF < 20%	COAPT‐excluded	4 (30.7%)
Inotropes	COAPT‐excluded	5 (38.4%)
Procedure duration (min)	162.9 ± 118.1	188 (164.2–203.5)
MR characteristics	Mainly central regurgitant jet (A2/P2)	Eccentric jets in 23% of cases

*Note:* Data are presented as absolute numbers and percentages (for categorical variables) and median value and 25th and 75th percentiles (for continuous variables).

Abbreviations: EF, ejection fraction; FAC, fractional area change; LVESD, left ventricular end‐systolic diameter; M‐TEER, mitral transcatheter edge‐to‐edge repair; MR, mitral regurgitation; RV, ventrículo direito.

**Table 6:** MitraClip devices used in each patient.

Patient	Mitraclip device
1	NTR (1)
2	XTR (2); NTR (1)
3	XTW (2)
4	XTW (1)
5	XTW (2)
6	XTW (1)
7	XTW (1)
8	XTW (2)
9	XTW (2)
10	XTW (2)
11	XTW (1)
12	XTW (1)
13	XTW (1)

Abbreviations: NTR, MitraClip NT regular; XTR, MitraClip XT regular; XTW, MitraClip XT wide.

The decision to pursue mitral valve repair is necessarily a multidisciplinary process in which the advanced HF cardiologist, the interventionist, and the echocardiographer must carefully evaluate all clinical and anatomical variables—including ventricular geometry, leaflet tethering, pulmonary pressures, and frailty—to determine whether a transcatheter approach is appropriate and technically feasible. Also, careful anesthesia planning is important to minimize deterioration during prolonged procedures.

Although most patients experience immediate hemodynamic improvement, a subset may develop transient low cardiac output after MR reduction and require temporary inotropic support or mechanical circulatory assistance. This phenomenon, known as AM, arises when abrupt elimination of the low‐resistance regurgitant outflow into the left atrium acutely increases effective LV afterload, reduces stroke volume, and—in patients with limited contractile reserve—leads to an immediate decline in LVEF with resultant hemodynamic instability [23]. AM has been associated with higher in‐hospital mortality among patients with LVEF < 35%, and individuals with proportionate MR appear particularly susceptible to this phenomenon, whereas preprocedural optimization with levosimendan and intensified diuretic therapy has been reported to exert a protective effect [24].

Given this, we believe that cautious, partial correction of MR may be safer than aggressive elimination in advanced HF, particularly in patients with severely dilated ventricles, very low LVEF (< 20%), or inotropic dependence. Reflecting this strategy, most patients did not achieve complete elimination of MR; grade 1 or 2 residual MR was intentionally accepted.

One of the most striking findings of this study is the high proportion of patients who were successfully weaned from inotropic support. It is important to emphasize, however, that discontinuation is not immediate, as some patients must first overcome the hemodynamic perturbations associated with AM. Moreover, based on our experience, inotrope withdrawal should be performed slowly and progressively—reflected by the median 9‐day weaning period in this cohort—to minimize the risk of acute decompensation.

Notably, no clinically overt episodes of AM were observed in this cohort. Such events may have been prevented, or their clinical expression attenuated, by the meticulous periprocedural management strategy implemented. As outlined above, this approach included careful anesthetic planning, strict intravascular volume control, temporary withdrawal of oral vasodilators, selective use of inotropic support (either transient periprocedural administration or continuation of pre‐existing therapy), gradual postprocedural inotrope weaning, and intentional acceptance of mild residual MR (grade 1–2) to avoid abrupt increases in LV afterload. Collectively, these measures may have contributed to the hemodynamic stability observed in this high‐risk population.

Taken together, our findings support the feasibility and potential clinical value of M‐TEER as a stabilizing strategy in carefully selected patients with advanced HF, particularly when performed by experienced multidisciplinary teams, and underscore the need for prospective studies to better define anatomical, hemodynamic, and clinical predictors of benefit in this high‐risk population.

## 5 Limitations

This study is a small, single‐center cohort, and the findings are derived from a highly selected population with advanced, refractory HF, which may limit the external validity and generalizability of the results. The severity of illness in this cohort—reflected by frequent inotropic dependence, severely depressed ejection fraction, and complex mitral valve anatomy—underscores the high‐risk nature of the population, but also introduces potential selection and referral biases. Furthermore, the favorable outcomes observed cannot be attributed solely to the M‐TEER procedure itself, but rather reflect the combined impact of a well‐trained multidisciplinary team, including interventional cardiologists, HF specialists, anesthesiologists, and imaging experts, working in a highly specialized center. This level of expertise and infrastructure may not be readily available in other settings, especially in resource‐limited environments.

## 6 Conclusion

In this initial experience, the MitraBridge strategy proved to be safe and effective in our cohort. Clinical outcomes were comparable to those reported in the original MitraBridge registry, despite the advanced clinical profile and anatomical complexity of our population. In a context where access to LVADs is severely limited and transplant waiting times are prolonged, M‐TEER emerges as a valuable therapeutic option to stabilize patients, bridge them to candidacy or transplantation, or even serve as DT in selected cases.

## Ethics Statement

This study was approved by the research ethics committee of Hospital de Clínicas de Porto Alegre.

## Conflicts of Interest

The authors declare no conflicts of interest.
